# Inflammatory Breast Carcinoma in Pregnancy: A Curious Case of a High-Grade Invasive Ductal Carcinoma Masquerading as a Breast Abscess in the Second Trimester

**DOI:** 10.7759/cureus.68984

**Published:** 2024-09-09

**Authors:** Navin Sundar Arunachalam Jeykumar, Subash Sacratice, Thivagar Thirugnanam, Pandiyan Vadivel

**Affiliations:** 1 General Surgery, Thanjavur Medical College Hospital, Thanjavur, IND

**Keywords:** breast abscess, high-grade invasive ductal carcinoma, inflammatory breast carcinoma in pregnancy, multidisciplinary management, triple-positive breast cancer

## Abstract

Inflammatory breast carcinoma is an uncommon presentation of carcinoma breast characterised by tumour cell emboli invading the dermal lymphatics and manifesting as skin oedema and redness, closely resembling acute mastitis. We report the case of a 37-year-old pregnant female in the second trimester (at 16 weeks) with a left breast inflammatory carcinoma deceptively presenting as a breast abscess leading to a late diagnosis, delayed definitive management, and having profound psychological, therapeutic, and prognostic implications. Given its tendency to be clinically misleading and often disregarded until late stages, much emphasis has to be placed on a low threshold of suspicion when suggestive clinical signs are encountered.

## Introduction

Inflammatory breast cancer is an uncommon and severe form of invasive breast cancer, characterised by erythema and edema involving a third or more of the skin of the breast and distinguished by rapid disease progression, both localized and distant metastases, earlier onset, and poorer overall prognosis compared to other breast cancer subtypes. Due to its infrequent occurrence, inflammatory breast cancer is frequently mistaken for acute mastitis or generalized skin inflammation. Breast carcinoma mimicking an abscess is encountered rarely and accounts for less than 1% of breast carcinomas [1]. Breast cancer is the most prevalent malignant tumour diagnosed during pregnancy, affecting approximately one in three thousand gestations [2]. Owing to the poor prognosis of breast carcinoma diagnosed during pregnancy due to the increased incidence of nodal metastasis, a high index of suspicion is required to initiate early management.

## Case presentation

We report the case of a 37-year-old female who had come to the emergency department with complaints of heaviness, swelling, and pain in her left breast for about 20 days duration. The patient was in the second trimester of pregnancy at 16 weeks and was having regular, scheduled antenatal check-ups at the local hospital with no known co-morbid illnesses. She gave us no history of nipple discharge, fever, weight loss, trauma, or backache and had no family history of breast cancer. She belonged to a lower socio-economic background and was a housewife with a 10-year-old son whom she had breastfed for two years.

**Figure 1:**
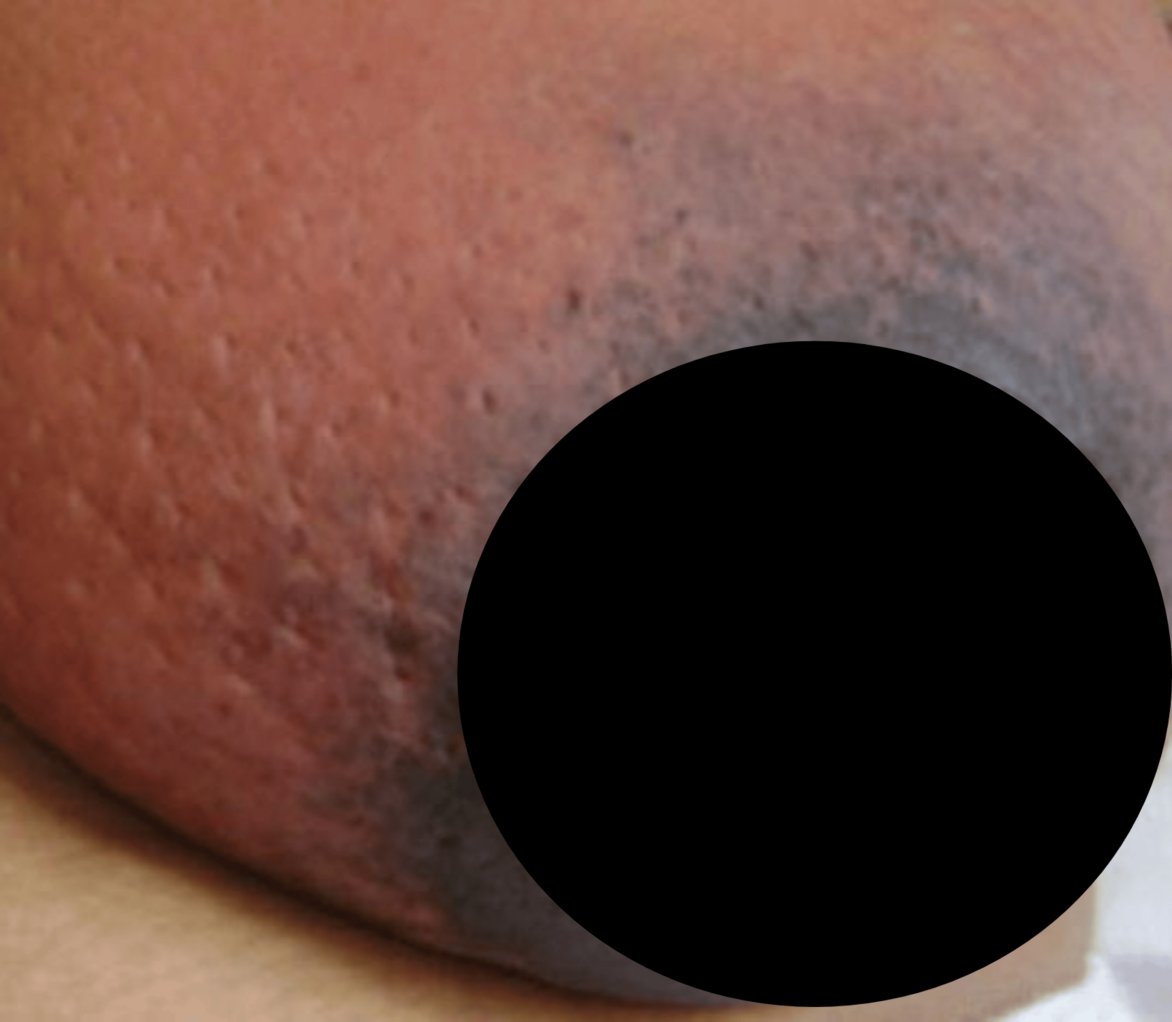
Redness and peau d'orange appearance over the upper and medial aspects of the left breast upon presentation.

On examination, her left breast was diffusely enlarged with redness and peau d’orange appearance observed over most of the upper hemisphere and the medial aspect (Figure 1). Warmth and tenderness were diffuse, and the upper quadrants of the breast felt firm and indurated to palpation with a soft, fluctuant area in the upper-inner quadrant. However, there was no skin ulceration, dilated veins, or a palpable lump and the nipple-areolar complex was normal. Axillary palpation revealed about three firm, non-matted, non-tender anterior, and central group of lymph nodes on the left side. No left supraclavicular nodes were palpable and the right breast and axilla were normal with no significant findings on systemic examination.

**Figure 2:**
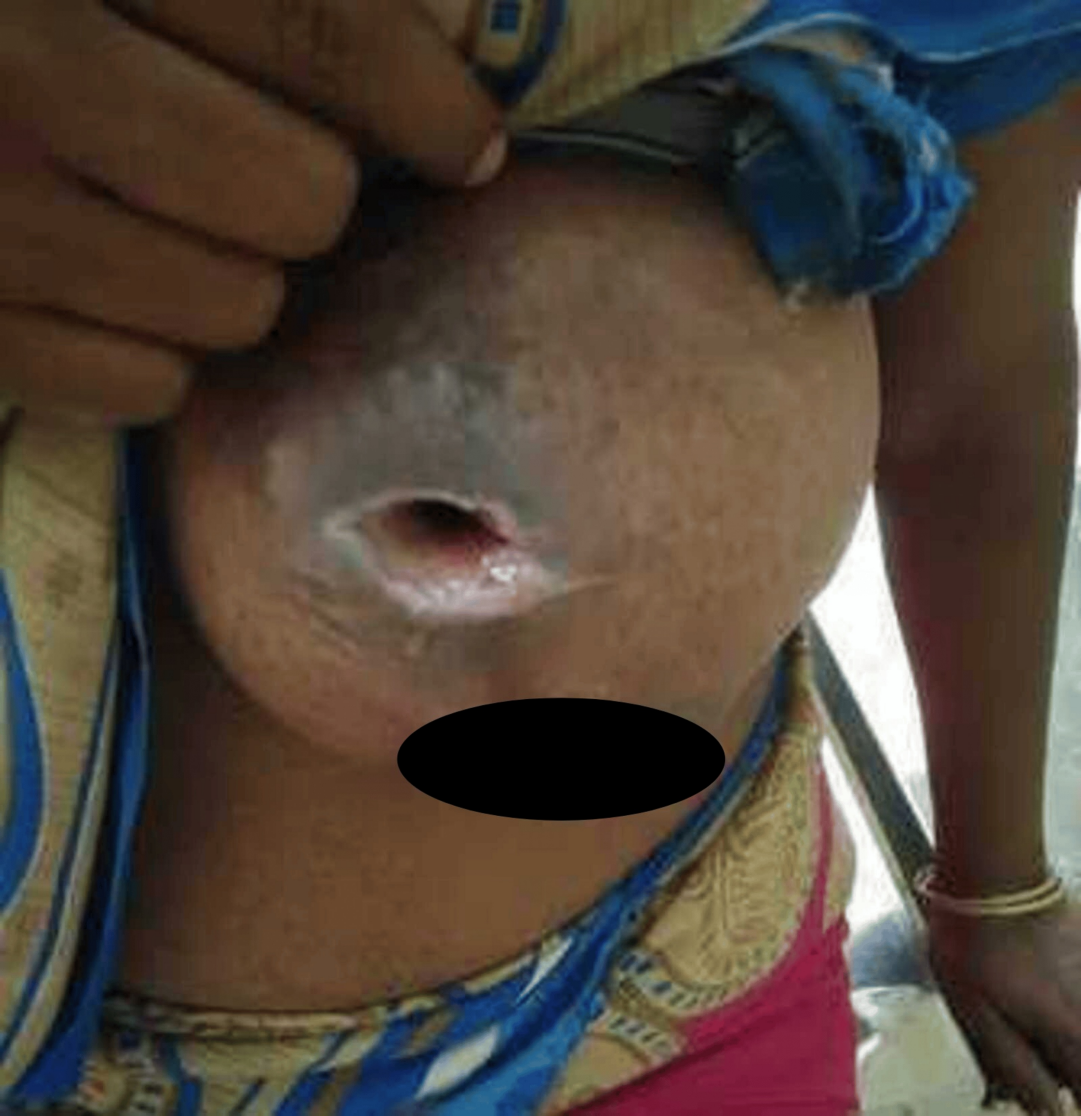
First follow-up at 1 week showed an open wound and poor healing with sero-purulent discharge.

An ultrasonogram revealed a 4 x 4 cm hypoechoic hemispherical cavity with thickened walls and posterior acoustic enhancement in the upper hemisphere of the left breast without internal debris and a provisional diagnosis of a breast abscess was made. The same day, the patient underwent an emergency incision and drainage in the upper inner quadrant under general anaesthesia, evacuating approximately 75 ml of greyish-brown sero-purulent discharge. The cavity was felt to be relatively small (4 x 5 cm) compared to the size of the engorged breast with indurated walls and no internal septation for which we saw no need to place a dependent drain tube. The discharge fluid was sent for bacterial culture and sensitivity and a piece of tissue was excised from the cavity wall and sent for histopathological study. The patient was empirically started on parenteral cefotaxime and analgesics. She was discharged with an open wound on the 4th post-operative day with oral cefixime and was asked to come for periodic check-ups. On the 7th post-operative day, the breast still appeared enlarged with a mild decrease in the local inflammatory signs (Figure 2). The surgical wound was still open and draining profuse sero-purulent discharge. Bacterial culture had reported normal skin commensals and the patient was asked to continue oral antibiotics and to be reviewed after a week. Histopathology showed non-specific descriptive findings related to chronic inflammation.

**Figure 3:**
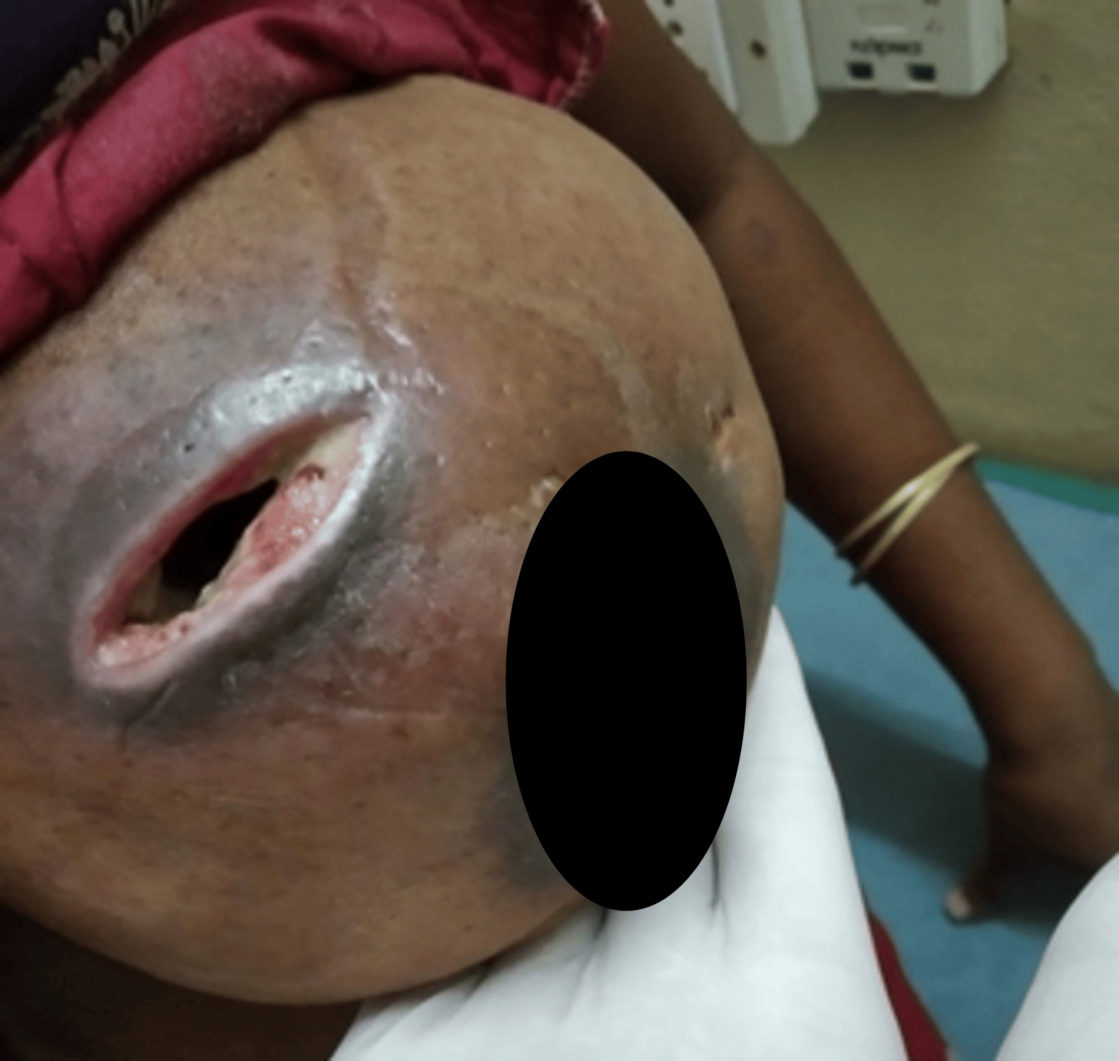
Second follow-up at 1 month demonstrated further swelling, induration and aggravated local inflammatory signs with poor wound healing and persistent wound discharge.

But the patient came for a review after one month with a still enlarged and painful breast, poor wound healing, and persistent sero-purulent discharge despite having been on oral antibiotics (Figure 3). Two tissue bits were incised from both the edge and just below the edge of the ulcer for histopathological study and she was restarted on empirical higher oral antibiotics (linezolid 600 mg twice daily).

**Figure 4:**
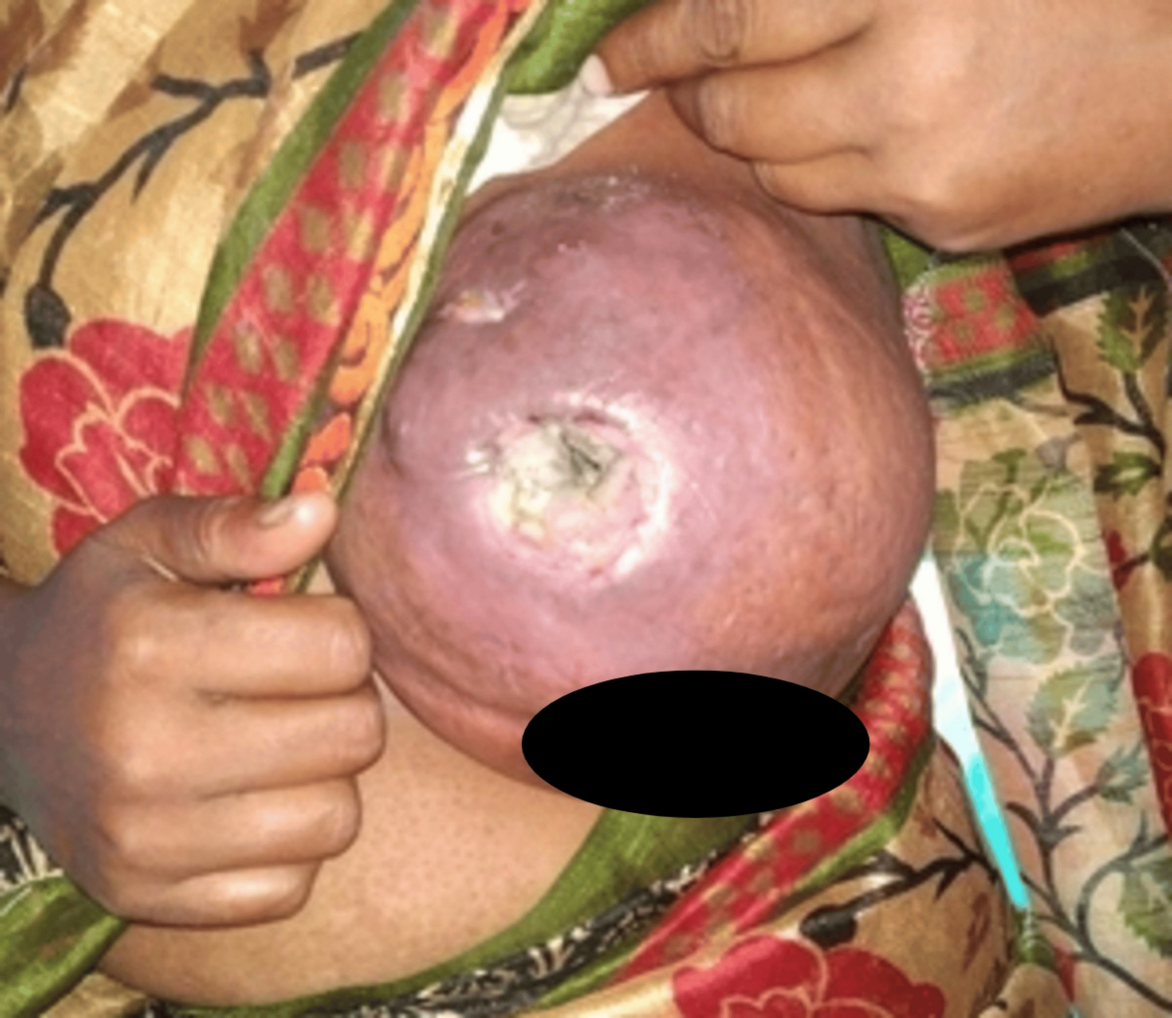
Third follow-up at 2 months showed a fungating mass lesion growing out of the surgical site with foul-smelling sero-purulent discharge and obvious skin inflammation and oedema.

Although instructed to have weekly consultations for regular follow-up, the patient came back one month after her second visit. On examination, the left breast had enlarged further with overt signs of cutaneous inflammation and a fungating, foul-smelling tumour mass had appeared at the surgical site entirely obliterating the abscess cavity (Figure 4).

**Figure 5:**
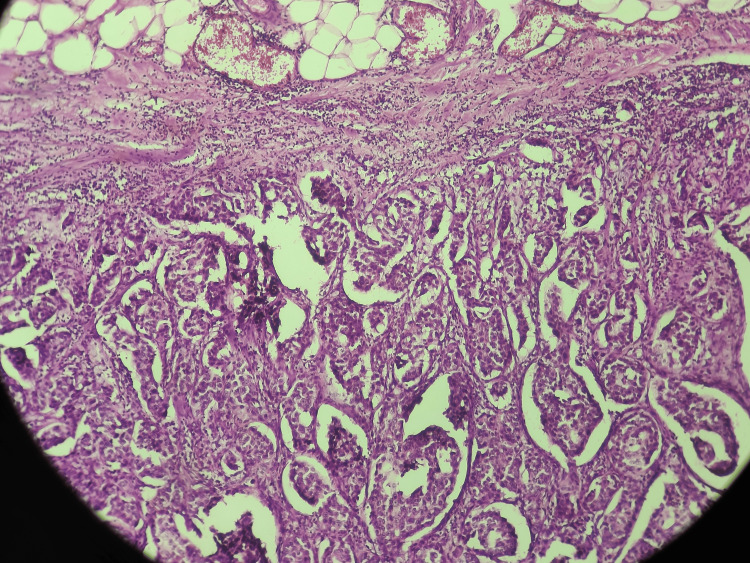
High-grade invasive ductal carcinoma with nesting pattern and lymphovascular invasion. Sheets and occasional tubules of malignant ductal epithelial cells within in a desmoplastic stroma. The tumor cells have pleomorphic, hypechromatic nuclei and prominent nucleoli.

The second biopsy reported high-grade invasive ductal carcinoma, no special type, with a nesting pattern and lympho-vascular invasion (Figure 5). Immunohistochemistry showed estrogen receptor (ER) and progesterone receptor (PR) positive, human epidermal growth factor receptor 2 (HER2)/neu 3+ (positive), and a Ki-67 index of 40%. A clinical diagnosis of inflammatory carcinoma of the left breast was made although pathognomonic histological findings like dermal lymphatic tumour emboli were not reported.

The patient and her husband were informed of the diagnosis and the available treatment options. An ultrasonogram showed diffusely increased, ill-defined tissue density with skin thickening over most of the left breast with a hypoechoic mass in the upper-outer quadrant and multiple non-matted hypoechoic left axillary lymph nodes, highly suspicious of metastatic tumour deposits. The right breast and axilla were normal. An ultrasound-guided fine needle aspiration cytology of the axillary nodes confirmed poorly differentiated adenocarcinomatous deposits. At a tumour board discussion, it was decided to advise medical termination of pregnancy in spite of the second trimester and start her on neoadjuvant chemotherapy. Before the decision for elective abortion was made, we took into consideration her age at 37 years, the biological aggressiveness of the tumour, the contra-indication to anti-HER2/neu drugs in pregnancy, the considerations of major non-obstetric surgery in the third trimester in higher maternal age, and importantly, the possibility of non-compliance or defaulting on chemotherapy due to a general lack of understanding, psychosocial stigma, and financial constraints. The treatment plan involved neoadjuvant chemotherapy and targeted therapy (anti-HER2/neu) followed by a modified radical mastectomy and adjuvant chemo-radiotherapy, targeted therapy, and endocrine therapy. The patient and her husband underwent psychological counselling and subsequently, she underwent medical termination of pregnancy by dilation and evacuation at 25 weeks of gestation. A full-metastatic imaging work-up was done and contrast-enhanced CT chest and abdomen films revealed no findings of distant secondary deposits. The proposed chemotherapy regimen included adriamycin, cyclophosphamide, paclitaxel, and anti-HER2 therapy. However, after completing one cycle of chemotherapy, she defaulted and was lost to follow-up.

## Discussion

The incidence of concurrent pregnancy among women with breast cancer ranges from 1% to 3% [3]. The average age of women diagnosed with breast cancer during pregnancy is the mid to late thirties [3]. Approximately 6% of women under 35 years old who are diagnosed with breast carcinoma are pregnant at the time of diagnosis [4]. Pregnancy-associated breast cancer (PABC) is defined as that which occurs either during pregnancy or within a year of childbirth. The incidence of PABC is notably elevated during the postpartum period compared to the gestational period prior to delivery. This can be attributed to the fact that masses detected during pregnancy may be perceived as normal physiological changes, leading patients to initially disregard them, while other symptoms like breast pain, skin and nipple abnormalities, and discharge can be attributed to breastfeeding leading to more locally advanced disease at the time of diagnosis [5].

PABC exhibits a highly aggressive biological profile, characterized by large tumour size, presence of nodal metastases, and high histological grade [6]. Existing research suggests that PABC is often linked to decreased levels of estrogen and/or progesterone receptors (83% cases) as well as lower HER2/neu expression [7-10], with other studies showing a higher expression of HER2/neu [11]. But one study has found no significant differences in hormone receptor and HER2/neu expression between PABC and non-pregnancy-associated breast cancer [12] and the HER2/neu overexpression was observed more in inflammatory (41%) than non-inflammatory cancers (19%) [13]. This was in contrast to the immunohistochemistry profile of our patient who showed a triple-positive receptor profile. Charafe-Jauffret et al. demonstrated that inflammatory breast cancer overall was defined by the following immunological profile: elevated E-cadherin levels, estrogen receptor negativity, high MIB1 proliferation index, cytoplasmic MUC-1 expression, and HER2 overexpression [14].

Similar to non-pregnant patients, most tumours (90%) are invasive ductal carcinomas and about 40% to 80% of patients have poorly differentiated cancers [15-17]. Research indicates that the prevalence of inflammatory tumours ranges from 1.5% to 4% [18], with some studies suggesting a higher incidence among pregnant individuals compared to non-pregnant controls [17].

A skin biopsy is not necessary to confirm the diagnosis when overt clinical signs of inflammatory carcinoma are present or when it is biopsy-proven. In one study, no pathognomonic dermal intralymphatic tumour emboli was observed in 50% of cases [13].

PABC has been linked to increased lympho-vascular invasion, more frequent lymph node metastases, and larger tumour size. Analogous to women who have never given birth, PABC typically spreads to the lung, liver, brain, and skeletal system with worse clinical outcomes and lower disease-free survival and a higher mortality rate compared to nulliparous women [9].

There is no epidemiological, clinical, or prognostic evidence suggesting that pregnancy or its termination alters the natural course of breast cancer or improves patient survival. Moreover, pregnancy itself does not necessarily compromise effective breast cancer treatment, although the selection and sequence of therapeutic modalities must be tailored for fetal safety. Treatment should be patient-tailored, depending on gestational age, staging at diagnosis, and patient preference. Broadly, surgical interventions in any trimester, chemotherapy in the second and third trimesters, and post-partum radiotherapy are considered safe and viable treatment options for most patients with pregnancy-associated breast cancer. However, for the few patients who present with advanced-stage disease during the first trimester, termination is typically recommended, as chemotherapy and/or radiotherapy at this stage are likely to pose a risk of fetal harm [19]. The National Comprehensive Cancer Network (NCCN) guidelines for breast cancer during pregnancy state that for a first trimester diagnosis of breast cancer, elective abortion has to be discussed with the patient. If she chooses to continue the pregnancy, the primary treatment would entail a modified radical mastectomy (MRM) followed by adjuvant chemotherapy in the second trimester with or without adjuvant radiotherapy and endocrine therapy in the post-partum period. In the second and early third trimesters, the mainstay is either upfront surgery (MRM or breast conservation surgery with axillary staging) with adjuvant chemotherapy or neo-adjuvant chemotherapy and surgery (MRM or breast conservation surgery with axillary staging) with or without post-partum adjuvant radiotherapy and endocrine therapy for both options. For the late third trimester, the recommendations are upfront surgery (MRM or breast conservation surgery with axillary staging) with adjuvant chemotherapy with or without post-partum adjuvant radiotherapy and endocrine therapy [20].

## Conclusions

Delays in breast cancer diagnosis during pregnancy are prevalent, and as a precautionary measure, all palpable lumps that appear suspicious should be thoroughly evaluated. The diagnosis of pregnancy-associated breast cancer poses a significant clinical management challenge for the pregnant patient. It is crucial to select treatment approaches that optimize the cancer outcome while minimizing fetal risk. Therefore, a multidisciplinary approach encompassing various medical disciplines is essential to attaining favorable outcomes, necessitating close collaboration among the breast surgeon, medical oncologist, clinical psychologist, and obstetrician with expertise in high-risk pregnancy management.
